# Editorial: Affective processing and non-invasive brain stimulation, volume II

**DOI:** 10.3389/fnhum.2023.1291645

**Published:** 2023-09-27

**Authors:** Delin Sun, Xiaochu Zhang, Jiawei Zhou, Nan Li

**Affiliations:** ^1^Brain Imaging and Analysis Center, School of Medicine, Duke University, Durham, NC, United States; ^2^Department of Veteran Affairs (VA) Mid-Atlantic Mental Illness Research, Education and Clinical Center, Durham, NC, United States; ^3^Hefei National Laboratory for Physical Sciences at the Microscale, University of Science and Technology of China, Hefei, Anhui, China; ^4^State Key Laboratory of Ophthalmology, Optometry and Visual Science and National Engineering Research Center of Ophthalmology and Optometry, Eye Hospital Wenzhou Medical University, Wenzhou, China; ^5^School of Mental Health and Psychological Sciences, Anhui Medical University, Hefei, Anhui, China

**Keywords:** affective processing, non-invasive brain stimulation (NIBS), event-related potential (ERP), transcranial magnetic stimulation (TMS), clinical

Affective processing plays a crucial role in human life. Deficits in affective processing are accompanied by mental problems including depression, anxiety, and addiction. Non-invasive brain stimulation methodologies such as transcranial magnetic stimulation (TMS) and transcranial direct current stimulation (tDCS) have become more and more popular in modulating a wide range of cognitive processing. This Research Topic aggregated several reviews, research articles, case reports, and research proposals aiming at identifying the neural correlates of affective processing and how these neural correlates are modulated by non-invasive brain stimulation.

## Neural correlates of affective processing

Quickly and accurately identifying facial expressions plays an important role in social interactions. Song et al. investigated the influence of emotional contextual information (fearful, happy, and neutral scenes) on the neural processing of fearful expressions during the early stages of facial recognition based on the face-specific N170 component derived from the event-related potential (ERP) method. Their findings suggest that people allocate more attention to the processing of facial information when the valence between emotional context and expression conflicts.

Although extensive research has focused on the detection of uncertain threat signals in anxious individuals, little has been done to investigate the detection of uncertain safety signals in people with anxiety. Jin et al. compared 16 subjects with high trait anxiety and 16 with low trait anxiety during a modified cue-target task in certain and uncertain stimulus blocks based on the ERP components P2 and N2 that reflect early attentional allocation and association learning, respectively. They reported distinctive attentional biases between high and low-trait anxiety individuals, as well as association learning in uncertain conditions in high-trait anxiety individuals.

Outcome evaluation plays an important role in reward learning and decision-making and is related to the benefits of both self and others. Tan et al. examined both behavioral and neural correlates of outcome evaluation by gambling for the self and charity, respectively. They employed two ERP components FRN and P3 to reflect the early and middle/late neural processing of outcomes, respectively. They reported that people tended to optimize strategies for themselves rather than for charity. Based on their ERP results, they concluded that people focus more on charity-outcome in the early stage, and more on self-outcome in the middle and late stages. The differences between self-outcome and charity-outcome varied with the reward magnitude. More specifically, people pay similar attention to small self-outcome and charity-outcome, but more attention to larger self-outcome than larger charity-outcome.

Cognitive reappraisal is one of the core treatment components of cognitive behavioral therapy and is the gold standard treatment for major depressive disorders. Wang et al. proposed a novel cognitive bias model that hypothesizes that cognitive reappraisal training may improve the generation ability of cognitive reappraisal with altered prefrontal–amygdala functional activation/connectivity, thus reducing negative cognitive bias (negative attention bias, negative memory bias, negative interpretation bias, and/or negative rumination bias) and alleviating depressive symptoms.

## Non-invasive brain stimulation and its effects

People who use methamphetamine for a long time may become less able to control their actions and more likely to act impulsively. Liu Q. et al. found that high-frequency repetitive TMS (HF-rTMS) on the left dorsolateral prefrontal cortex (DLPFC) is a promising intervention for reducing impulsivity and cue-induced craving in patients with methamphetamine use disorders. In their study, patients underwent five sessions of HF-rTMS on the left DLPFC per week for 4 consecutive weeks, while controls received no rTMS intervention.

Non-invasive brain stimulation methods are well-accepted in both academic and clinical domains focusing on not only affective processing but also the other cognitive processing. Qi et al. systematically reviewed the existing literature on the effects of tDCS on motor skills learning of healthy adults and discussed the underlying neurophysiological mechanism that influences motor skills learning. They included 11 studies in their meta-analysis and showed that tDCS can help healthy adults improve motor skills learning through activating different brain regions including the primary motor cortex, left DLPFC, and right cerebellum.

Cryptococcal meningitis is a central nervous system disease caused by a novel Cryptococcus infection that leads to subacute or chronic inflammatory changes in the nervous system. In a case study, Liu Y. et al. examined a 72-year-old woman diagnosed with Cryptococcal meningitis and severe cognitive impairment and disabilities. They reported a large improvement in cognitive functions, especially executive functions, after receiving anti-infectious and rTMS.

## Novel methods

Non-invasive brain stimulation should not be limited to TMS and tDCS methods. Adding noise to a system to improve a weak signal's throughput has been shown to improve sensory perception and even higher-order processing such as working memory. To understand whether stochastic resonance can broadly improve cognition, Sherman et al. investigated the performance of different cognitive tasks while applying auditory white noise and/or noisy galvanic vestibular stimulation. They found that some subjects exhibited cognitive changes with the addition of noise but the rest subjects did not. Their findings suggest that using noise to improve cognition is not applicable to a broad population; however, the effect of noise differs across individuals.

Machine learning is very promising in clinical usage. In order to improve diagnosis in clinical patients by using the functional connection methods, Zhao et al. developed a new multi-view brain network feature enhancement method based on a self-attention mechanism graph convolutional network (SA-GCN). This method enhances node features through the connection relationship among different nodes, and then extracts deep-seated and more discriminative features. They reported that SA-GCN effectively extracts more discriminative features and achieves the best classification accuracy (79.9%) for the diagnosis of autism spectrum disorder. Their new methods have the potential to be applied to patients with other disorders including problems of affective processing.

In summary, our Research Topic may help to delineate the neural correlates of affective processing as well as the influences of non-invasive brain stimulations on neural processing including affective processing, and may help to propose potential empirical interventions for clinical disorders.

## Author contributions

DS: Conceptualization, Writing—original draft, Writing—review and editing. XZ: Writing—original draft, Writing—review and editing. JZ: Writing—original draft, Writing—review and editing. NL: Writing—original draft, Writing—review and editing.

